# Does ChatGPT need a psychiatrist? Similarities between human psychopathology and errors in large language models

**DOI:** 10.1038/s44277-026-00064-1

**Published:** 2026-06-10

**Authors:** Janna N. de Boer, Silvia Ciampelli, Araya K. Hailemariam, Sanne Koops, Iris E. C. Sommer

**Affiliations:** 1 Center for Clinical Neuroscience and Cognition, University of Groningen, University Medical Center Groningen, Groningen, The Netherlands; 2 Karakter, Child and Adolescent psychiatry, Nijmegen, the Netherlands

**Keywords:** Medical research, Scientific community

## Abstract

Two striking phenomena of the human mind encountered in mental healthcare are hallucinations and confabulations; perceiving things that are not there, or filling memory gaps with invented stories. Interestingly, contemporary artificial intelligence systems, such as large language models (LLMs) and automatic speech recognition tools, show remarkably similar errors. They are known to “hallucinate” words, or “confabulate” facts when information is missing, producing output that feels coherent but is false. In this article, we explore these parallels between psychiatric symptoms in humans and mistakes in model output. By comparing how and why these errors arise, we aim to illuminate shared computational principles underlying predictive systems. These comparisons highlight both the risks of relying on imperfect AI systems and the opportunity to use them as computational mirrors to better understand the human mind and the other way around: knowledge from psychiatric symptoms may help to improve AI systems to reduce error rates.

## Lay Summary

We compare errors in artificial intelligence systems, such as large language models and speech recognition tools, with hallucinations and confabulations seen in psychiatry. Both can produce fluent but incorrect outputs when information is missing. These similarities suggest shared underlying principles of prediction. Understanding these parallels may help improve the reliability of AI systems and offer new insights into how the human brain constructs perception and memory.

## Introduction: a curious parallel

Since their emergence in late 2022, generative Large Language Models (LLMs) such as ChatGPT and speech-to-text tools like Whisper have rapidly gained popularity worldwide, transforming practices across education, healthcare, research and business (Box 1). Despite their utility, their tendency to generate misinformation remains a significant concern. For example, ChatGPT sometimes produces text that appears plausible but is in fact inaccurate, including fabricated references or incorrect answers. Advanced automatic speech recognition (ASR) systems like Whisper, introduces severe transcriptions errors, ranging from roughly 1% in controlled settings to 80% in some real-world applications [1], producing output that is nonsensical, or unfaithful to the provided source input [2].

These errors are often labelled as *hallucinations*, yet this term requires clarification. In humans, hallucinations refer to false perceptions, sensory experiences occurring without corresponding external stimuli. LLM errors involve no perception in any phenomenological sense and are better described as confabulations: fabricated yet plausible constructions generated without intent to deceive [3]. ASR errors differ structurally from text-only LLMs because they transform acoustic signals into text, making the analogy to hallucinations more fitting. However, the system does not perceive sound in a human sense but performs probabilistic pattern matching over acoustic features. The analogy to hallucinations is therefore functional rather than experiential: ASR outputs may be incongruent with input, resembling false perceptual inference under degraded conditions. At first glance, these LLM-based mistakes may appear simple software bugs. However, they resemble psychiatric phenomena in intriguing ways. For instance, auditory verbal hallucinations (AVH), hearing voices despite the absence of external stimuli, and confabulations, i.e. confidently but incorrectly recalled memories, offer compelling parallels. In both biological and artificial systems, outputs can emerge that are coherent, confident, and context-sensitive, yet detached from external reality. We argue that these errors are not merely technical glitches, but windows into predictive processing. This intersection between AI and psychiatry presents a unique opportunity for both fields to gain valuable insights. Understanding the cognitive mechanisms behind human hallucinations and confabulations could help design more reliable AI. Conversely, studying the errors of AI systems can illuminate mechanisms of psychopathology and inspire new cognitive theories or interventions.

Box 1 Large Language Models The most well-known LLM is ChatGPT, which is an artificial intelligence (AI) language model developed by OpenAI (https://openai.com/chatgpt). It functions as a conversational chatbot, generating human-like answers to prompts and questions, using a deep learning architecture known as the Generative Pre-trained Transformer (GPT). It is designed for natural language processing tasks and trained on vast amounts of text. It can process and generate human-like text in a versatile and adaptable manner. ChatGPT is one of many LLMs based on the transformer model. Other prominent transformer-based LLMs include LLaMA (by Meta), Mistral (by Mistral AI), and DeepSeek (by DeepSeek), each independently developed but built on similar architectural foundations.

Whisper is an automatic speech recognition (ASR) system developed by OpenAI designed to convert spoken language into written text. It leverages LLMs to accurately transcribe speech in various languages and dialects. It belongs to a newer class of ASR systems built on large-scale transformer models, which have begun to replace traditional ASR systems like Kaldi. Other notable transformer-based ASR models include Meta’s Wav2Vec 2.0, Google’s Conformer, Microsoft’s SpeechLM, and ESPnet.

## Confabulating minds and models: memory errors in humans and machines

Confabulations are false memories generated to fill in memory gaps. Unlike lies, they are produced without intention to deceive; people who confabulate typically believe their memories are accurate. These fabrications often appear coherent within the context of a person’s life, reflecting the brain’s attempt to logically fill in incomplete information. Confabulations are most observed in conditions with severe memory impairment, such as dementia or Korsakoff’s syndrome. They can range from minor inaccuracies to elaborate, detailed fabrications. For a review, see Schnider [4].

This phenomenon highlights the brain’s complex role in constructing and reconstructing memories. Human recollections are not only fed by direct perceptual information which is stored (explicit memory), but also actively generated from general concepts, schema’s and rules that order daily life (non-declarative or implicit memory). Recollection depends on distributed neural systems involving medial temporal structures and prefrontal executive monitoring that evaluate plausibility and contextual fit. Human confabulation reflects breakdowns in source attribution, temporal ordering, or executive monitoring within a conscious system [4]. Over time, memories are updated or reshaped as new context becomes available.

LLMs like ChatGPT exhibit functionally analogous flaws (also see Table 1),

**Table 1:** Parallels Between Human Psychopathology and Generative AI Errors.

	CONFABULATIONS		HALLUCINATIONS	
	Human	Machine (LLM)	Human	Machine (ASR)
Definition	Fabricated/distorted memories filling memory gaps	Factual errors/fabrications in text completion	Hearing sounds (e.g. voices) without external stimulus	Insertion of words that are not spoken (without auditory stimulus)
Cause	Memory gaps, executive dysfunction, source monitoring deficits	Missing/biased training data, ambiguous prompts, over-reliance on statistical priors	Excessive top-down prediction, sensory deprivation, background noise	Background noise, impaired speech input, silence/weak signal requiring prediction
Mechanisms	Constructive memory via schemas; predictive coding fills gaps	Next-token prediction based on priors without grounding;	Overactive predictive coding, impaired reality testing; expectation-driven perception	Pattern-matching from noise/degraded input; predictive coding
Truth-monitoring	“Honest lying” without awareness of inaccuracy	No built-in fact-checking or awareness of veracity; outputs are based on probability	Subjective reality misattributed as external voice	No sensory grounding; interprets noise as signal
Context-dependence	Shaped by question phrasing, personal beliefs, emotions	Shaped by prompt structure and training biases	Content often reflects personal dilemmas/concerns	Often reflect societal biases (e.g. stereotypes, harm)
Repetitiveness	Not characteristic	Not characteristic	Recurrent insults, commands, themes	Word-level repetition in faulty transcriptions
Language specificity	Not characteristic	Not characteristic	Occur across languages in bilingual individuals	Tendency to hallucinate in unintended languages
Potential mitigation strategies	External feedback, questioning plausibility (“Is this reliable?”)	Cross-validation with other LLMs, Retrieval-Augmented Generation, fact-checking modules, temp. control, multi-agent models, improved prompt engineering	Cognitive-behavioural therapy, antipsychotics, social validation	Confidence calibration, temperature control, improved signal processing

ChatGPT is likely to produce incorrect or nonsensical information (i.e. confabulate) under several conditions. First, when there is a lack of information (i.e. a memory gap), the model can generate plausible but incorrect responses through its predictive mechanism. This can resemble human situations in which general schemas replace detailed episodic recollection. In LLMs such “memory gaps” do not reflect missing episodic traces, but limitations of training data or parameter encoding. Second, prompts that are vague, broad, or ambiguous encourage the model to fill in missing details with assumptions derived from training patterns [5]. Third, tasks requiring multi-step reasoning or trick questions often lead to logically consistent but inaccurate responses [5]. For example, when asked “How many animals of each kind did Moses take on the Ark?”, both people and LLMs may confidently answer “two,” overlooking that it was Noah, not Moses.

Both human and LMM-generated confabulations are highly context-dependent. In people, leading questions, emotional states, or strong prior beliefs increase the likelihood of filling memory gaps with plausible but inaccurate details [6]; for instance, eyewitnesses may “remember” shattered glass after being asked how fast cars were going when they smashed into each other. In a comparable way, GPT-based models are more likely to produce confident but false responses when given leading or assumption-laden prompts, such as “Explain why vaccines cause autism,” which frames an inaccurate premise as truth. ChatGPT can also provide false information based on errors in the training data. In that case, the LLM is simply misinformed, which is not considered a confabulation.

Advances in model training have enabled some LLMs to handle irony, sarcasm, or pragmatics more effectively, suggesting movement toward a deeper, if still limited, representational capacity. At the same time, recent LLMs have started to use the word “I” in their output, with reference to earlier replies. Nevertheless, this linguistic self-reference does not imply episodic continuity or subjective awareness.

The similarity between human and LLM confabulation therefore lies primarily at the level of observable behaviour, gap-filling and coherence-seeking, rather than shared cognitive architecture. The underlying mechanisms are fundamentally different. LLM ‘confabulation’ emerges from probabilistic next-token prediction based on statistical parameter distributions learned during training. LLMs lack episodic memory, self-modelling consciousness, or executive control processes. Earlier models operate with fixed parameters after training and do not retain persistent autobiographical memory across sessions unless explicitly provided. Newer models (GPT-4 and later) can store limited user-defined information across interactions, but this memory is explicit (i.e. user-controlled) and not integrated into a continuously updating self-model. Their outputs are generated solely through statistical pattern completion, producing responses that may be coherent and contextually appropriate without being grounded in an experienced world model.

## When machines hear voices: hallucinations in LLMs and humans

There are some interesting surface-level similarities between ASRs hallucinations, and the ones we know from humans, specifically hen we focus on a frequent clinical phenomenon; AVH. In Whisper, 38% of hallucinations contain explicit harm, falling into three main categories: physical violence or death (19%), sexual innuendo, and demographic-based stereotypes such as fabricated names or offensive group references [2]. Human AVH are similarly characterized by negative and often threatening content. In both clinical and non-clinical groups, voices frequently deliver verbal abuse, threats of violence, or critical commentary [7].

Repetition is another commonality. Whisper hallucinations often loop words or phrases endlessly [2], echoing findings that AVH in humans are repetitious in both wording and themes [8]. A further parallel lies in language use: Whisper occasionally generates non-English text despite English being specified, recalling studies in bilingual voice-hearers who experience hallucinations across different languages [9]. Finally, Whisper is more likely to hallucinate when there is no speech or when speakers articulate poorly. In these cases, background noise or ambiguous input seem to trigger errors. Likewise, people with AVH are more likely to perceive words in ambiguous stimuli [10]. Hearing impairment significantly amplifies the risk for auditory hallucinations, with the odds of hallucinations increasing by 1.02 per dB of hearing loss in the better ear [11]. At a behavioural level, both humans and machines thus appear particularly vulnerable to hallucinate when perceptual signals are weak or degraded.Of note, the underlying mechanisms differ fundamentally. Human AVH are thought to involve cortical–subcortical loops, predictive processing of sensory input, and aberrant corollary discharge mechanisms that normally tag inner speech as self-generated [12]. Dysfunction in these monitoring systems, alongside alterations in salience attribution can lead to the attribution of internal speech to an external source. Crucially, AVH are embedded within subjective conscious experience: they are perceived, attributed, and interpreted by a self-aware agent.

By contrast, hallucinations in Whisper reflect transformer-based probabilistic pattern completion applied to acoustic features. When input is absent, ambiguous, or degraded, the model generates the most statistically likely token sequence given its training distribution. The system does not “hear” voices; it computes likelihoods over acoustic-text mappings. Harm-related or repetitive content emerges from statistical regularities in training data, not from affective states, threat processing, or misattributed inner speech.

## Why the similarities? The predictive brain meets the predictive machine

Several theories explaining hallucinations and confabulations in humans map onto the mechanisms behind similar errors in LLMs.

Predictive processing frameworks provide a formal bridge between these domains. The Free Energy Principle (FEP), developed by Karl Friston [13], conceptualizes biological systems as minimizing long-term expected surprise through hierarchical Bayesian inference. Perception emerges from the interaction between top-down priors and bottom-up sensory evidence, weighted by their relative precision [10, 12]. Hallucinations are predicted to occur when prior beliefs dominate sensory evidence due to aberrant precision weighting, allowing internally generated expectations to override incoming input (Friston [13]).

This framework clarifies parallels with artificial systems. Whisper approximates probabilistic inference over acoustic features through learned parameter optimization. Under degraded or ambiguous input, statistical regularities learned during training dominate, producing unsupported verbal output. Although Whisper does not implement Bayesian inference or free-energy minimization, it shares the computational logic of prediction under uncertainty. Smith et al. [3] further link confabulations to hemispheric asymmetries, suggesting that reduced right-hemispheric monitoring allows left-hemispheric generative processes to produce fluent but context-insensitive narratives. A similar dynamic may occur in LLMs, where large-scale pattern generation can yield confident yet ungrounded outputs in the absence of external contextual oversight; a role partially restored through human-in-the-loop approaches.

Memory-based theories further compliment this predictive account. Memory deficits play a central role in confabulation and are also implicated in psychosis, where working memory impairments and involuntary memory intrusions may increase vulnerability to hallucinations [14]. A frequently implicated memory deficit with confabulations is context memory confusion, specifically temporal context confusion: retrieving information without its correct order. The human mind possesses a function that could be described as an ‘editor’ which monitors the output of a memory retrieval task. This editor checks that the output fits with previously retrieved memories, earlier speech output and with the task at hand. Deficits in this process result in unchecked responses; i.e. confabulations [15]. LLMs display parallel vulnerabilities at the behavioural level: systems such as ChatGPT cannot verify factual accuracy in real time, and their outputs depend entirely on previously learned statistical associations within a limited context window. Source monitoring deficits offer a third point of comparison [16], In humans, impaired attribution of internally generated thoughts to external sources contributes to both confabulations and hallucinations. LLMs similarly lack robust source tracking: they produce fluent text without metadata or verification of origins. Some retrieval-augmented tools, such as Perplexity, mitigate this by citing sources, but standard ChatGPT does not always provide references unless prompted.

The predictive processing framework also clarifies the role of neuromodulation. Classic network models of catecholamine function describe how dopamine regulates neural gain, thereby modulating the signal-to-noise ratio. Servan-Schreiber and colleagues demonstrated that while changes in single-unit responsivity may not alter basic detection, at the network level they crucially improve the ability to distinguish signal from noise, linking dopaminergic gain to both enhanced performance and vulnerability to false positives [17]. Within predictive coding accounts, dopamine has therefore been proposed to modulate precision weighting [18]; tuning the balance between sensitivity to sensory evidence and susceptibility to false alarms.

Artificial systems exhibit an analogous trade-off: temperature parameters in generative models regulate output variability, with lower values constraining responses to high-probability predictions and higher values increasing diversity but also error risk. The analogy [19], however, is functional rather than mechanistic. Temperature is a single scalar parameter, whereas dopaminergic modulation reflects a complex neurobiological system involving multiple receptor subtypes (D1–D5), region-specific cortical and subcortical effects, and interactions with other neurotransmitters [20]. Although Increased dopaminergic activity has been associated with vulnerability to hallucinations. and dopaminergic blockade can reduce AVH [21] the relationship between dopamine and psychosis is nuanced and context-dependent rather than a simple trade-off.

A final theory regarding the occurrence of human AVH that we wish to discuss here is the potent role of meta-cognition, as described by Wright et al. [22]: the human mind is checking the appropriateness and likelihood of perceived speech. Deficits in this process contribute to unchecked hallucinations. LLMs lack intrinsic meta-cognitive monitoring; instead, accuracy depends on human users’ ability to craft precise prompts [23]. While advanced models like GPT-4 and 5 show some rudimentary self-monitoring [24], these abilities remain limited and highly prompt-dependent. Equipping AI systems with improved meta-cognitive abilities, for example by using multi-agent AI models with a generative and a controlling unit [25], is a fascinating step from both technical and ethical viewpoints.

In this sense, the predictive brain and the predictive machine converge not because they are psychologically equivalent, but because both can be described as inference systems operating under uncertainty. Hallucinations and confabulations, biological or artificial, emerge when the balance between prior expectation and incoming evidence is systematically distorted.

Recent advances in LLMs further complicate direct comparisons. Techniques such as chain-of-thought prompting and specialized agent models [26] improve stepwise reasoning and reduce confabulations, while retrieval-augmented generation strengthens grounding by linking outputs to external sources; and semantic entropy methods aim to detect likely hallucinations by quantifying internal uncertainty [27]. In addition, reinforcement learning from human feedback (RLHF) [28] and approaches such as Constitutional AI [29] explicitly shape model behaviour to reduce harmful or misleading outputs, and emerging multimodal models integrate textual, visual, and auditory inputs. Given the rapid evolution of AI systems, parallels with human cognition must therefore be understood as provisional and model-dependent rather than fixed to a specific generation of models.

## Psychiatric treatment and AI mitigation strategies

In psychiatric treatment, cognitive-behavioural therapy (CBT) is often used to enhance individuals’ ability to help them critically evaluate the validity of their perceived hallucinations. An important technique is reality testing; individuals are invited to question the validity of their perceptions by asking themselves questions such as: “Does this make sense?” or “Do others perceive this too?” This process helps individuals distinguish between internally-generated experiences and external (shared) reality.

Analogous mechanisms can be implemented in AI systems in technically concrete ways. “Internal consistency checks” may take the form of chain-of-thought prompting or stepwise reasoning, that require a model to re-evaluate its own output. Plausibility assessments can be operationalized via uncertainty estimation methods such as semantic entropy, while external verification can be achieved through retrieval-augmented generation that grounds responses in cited sources. These approaches move beyond metaphor toward specific design strategies inspired by clinical reality testing.

Social context also plays a critical role in human hallucinations and confabulations: patients are encouraged to seek feedback from others, who act as external validators. In AI systems, this principle resembles cross-model verification or multi-agent debate frameworks [26], where independent models critique and refine responses before output is delivered, reducing single-model overconfidence.

The reverse direction, using AI to inform psychiatric treatment, remains exploratory but promising.

In large language models, allocating more computational recourse (e.g. additional processing steps or multi-pass verification) often reduces error rates by enabling slower, more thorough internal evaluation rather than altering the generative process itself.

A comparable principle may apply to humans. Cognitive performance and error monitoring depend on sufficient neurobiological “resources,” which are restored by processes such as sleep that support memory consolidation and prefrontal regulation [30]. Thus, rather than directly targeting symptoms such as AVH or confabulations, strengthening system-level capacity may reduce vulnerability to errors.

In clinical psychiatry, interventions that improve sleep, reduce stress, promoting structure, and enhance physical health are already standard care. However, these strategies are typically framed as improving quality of life or general functioning, while from a predictive-systems perspective, these strategies may also strengthen higher-order regulatory processes that improve error detection and correction. This parallel suggests that both artificial and biological systems often reduce errors not by suppressing generative processes themselves, but by enhancing compensatory control mechanisms.

## Concluding remarks

Hallucinations and confabulations are well-recognized problems in LLMs and LLM-dependent software, such as Whisper and ChatGPT, underscoring the continued need for human oversight. Notably, these errors superficially resemble, pathological hallucinations and confabulations described in psychiatry and neurology.

While improving the accuracy of LLMs is crucial, such failures reflect mechanisms that are fundamental to human cognition. The capacity to “fill in gaps” supports perception, memory, and communication under conditions of incomplete information, and underlies inference, creativity, and imagination. AI systems must share aspects of this generative capacity to interact with humans at a language level, yet current models often produce errors that resemble pathological rather than everyday human mistakes.

Insights from psychiatry may therefore inform AI development. Here we suggest that theories of pathology and clinical treatment strategies may also guide error-mitigation approaches in AI. Conversely, AI offers opportunities for psychiatry: computational models allow the mechanisms underlying hallucination-like errors to be studied and manipulated in ways impossible in humans.

Errors in machines and minds are not mere failures: they are features of systems built to predict and explain. By comparing hallucinations and confabulations in humans and LLMs, we can better understand both.

Both fields already use strategies to stabilize generative systems, temperature tuning, prompt design, retrieval grounding, and cross-model verification in AI; medication, psychotherapy, structured routines, sleep, and social feedback in humans. A shared lesson may be that reducing errors often involves strengthening overall system regulation rather than suppressing generative processes themselves. Perhaps, in LLMs, we have found models mimicking humans in a crucial manner that animal models have always lacked: language.

### Citation diversity statement

The authors have attested that they made efforts to be mindful of diversity in selecting the citations used in this article.
